# Strategic HRM and SME innovation: a chain mechanism of learning-resilience pathway and nonlinear environmental dynamism

**DOI:** 10.3389/fpsyg.2025.1584489

**Published:** 2025-06-10

**Authors:** Jiabao Wang, Jiaxue Zhang, Yi Zhao

**Affiliations:** School of Management, Shanghai University, Shanghai, China

**Keywords:** SHRM, organizational innovation (OI), organizational learning (OL), organizational resilience (OR), environmental dynamism, SMEs (small and medium enterprises), dynamic capabilities (DC)

## Abstract

In an increasingly competitive business environment, organizational innovation has become crucial for enterprises seeking to establish and maintain a competitive advantage. This study aims to investigate how SHRM drives innovation in SMEs through the chain mediation of organizational learning and resilience, while exploring the nonlinear moderating role of environmental dynamism. Grounded in dynamic capability theory, we propose a three-dimensional framework that integrates internal organizational mechanisms with external environmental contingencies. Utilizing a three-wave survey design with 256 technology-focused SMEs in China’s Yangtze River Delta region, we employed hierarchical regression analysis and bootstrap mediation tests to validate hypothesized relationships. The findings indicate that SHRM significantly contributes to promoting organizational innovation in SMEs. Organizational learning (as a knowledge-building process) and resilience (as an adaptive capacity) sequentially mediate this relationship, forming an internal “learning-resilience” mechanism in which knowledge acquisition fosters robust adaptation, thereby synergistically enhancing innovation capability development. Furthermore, environmental dynamism exhibits a significant inverted U-shaped moderating effect: moderate levels amplify SHRM’s innovation-enhancing effects, whereas excessive dynamism diminishes its efficacy, revealing a “dynamic-changing” boundary condition. Theoretically, this study advances dynamic capability theory by unraveling the synergistic interplay between internal capability-building processes and external environmental contingencies. Practically, this study provides actionable insights for SME managers to strategically allocate human resources, cultivate learning-resilience capacities, and adaptively respond to environmental turbulence, thereby fostering sustainable innovation in volatile markets.

## 1 Introduction

The contemporary business environment, characterized by profound changes and adjustments, has become increasingly complex, dynamic, and uncertain (Teece, 2007). Navigating this inherent environmental dynamism is a primary challenge for the sustained development of small and medium-sized enterprises (SMEs). Scholars are focusing on effective strategies to manage and respond to this uncertainty (Li et al., 2024; Sarfo et al., 2024). Existing research emphasizes the critical role of ongoing organizational innovation in addressing business environment shifts, especially for SMEs. Due to their limited resources and capabilities, SMEs are more vulnerable to uncertain business conditions (Donbesuur et al., 2020; Klewitz and Hansen, 2014; Radziwon and Bogers, 2019). Thus, continuous organizational innovation is essential for SMEs to survive and grow. This study explores sustainable innovation pathway for SMEs and its evolution under environmental dynamism, which is central to our research.

Previous studies on SMEs’ organizational innovation have examined various perspectives such as strategic management (Salavou et al., 2004; Hervas-Oliver et al., 2014; Terziovski, 2010), organizational structure (Laforet, 2011, 2013; Radziwon and Bogers, 2019), resource allocation (Terziovski, 2010; Laforet, 2013), and contingency views (Popa et al., 2017; Ganter and Hecker, 2014; Ahmed et al., 2020) to identify pathways that enhance innovation performance. However, a notable gap exists, as current research often examines internal or external organizational factors in isolation, failing to capture the synergistic interplay between strategic human resource allocation and environmental contingencies. This fragmented perspective limits our understanding of how SMEs dynamically configure their internal capabilities to adapt to external turbulence. The dynamic capability view (DCV) addresses this gap by emphasizing the continuous coordination and integration of internal and external resources, enabling organizations to adapt to changing environments (Wilden et al., 2016). Enterprises with dynamic capabilities can navigate uncertain environments and achieve sustainable competitive advantages over time (Ferreira et al., 2020; Schilke et al., 2018).

Prior research has highlighted organizational learning and resilience as pivotal capabilities influencing innovation. Organizational learning refers to the institutionalized processes through which SMEs systematically acquire, interpret, and apply knowledge to reconfigure their operational routines (Argote and Ren, 2012; Flores et al., 2012). This capability enables the continuous adaptation of cognitive schemas of human capital, which is particularly crucial for SMEs operating in technology-intensive sectors (Cappellin and Wink, 2009). Conversely, organizational resilience embodies the dynamic capacity to anticipate disruptions, maintain functional integrity during crises, and reconfigure resources for post-shock recovery (Kantur and İşeri-Say, 2012; Lengnick-Hall et al., 2011; Tierney, 2003; Williams et al., 2017). In the context of SMEs, this manifests as strategic flexibility in human capital deployment to balance innovation risk and operational stability (Do et al., 2022). However, these capabilities often remain latent within human capital and require Strategic Human Resource Management (SHRM) practices to effectively allocate human resources and ignite employees’ passion, willingness, and capacity for innovation (Chen and Huang, 2009). Recent studies have demonstrated that SHRM’s emphasis on cross-functional teaming and ambidextrous reward systems can simultaneously strengthen learning agility and buffering capacity (Ho et al., 2023; Zhang et al., 2024). Specifically, developmental performance appraisals foster experimental learning cultures, whereas flexible job designs build adaptive resilience—dual mechanisms that are critical for the sustainability of SMEs’ innovation (Zahoor et al., 2020; Sarfo et al., 2024).

SHRM involves a series of planned activities aimed at leveraging human resources to achieve organizational objectives (Jackson et al., 2014). These practices encompass various components such as recruitment, training, employee participation, performance management, and compensation (Chen and Huang, 2009). The focus extends beyond attracting, developing, and retaining human capital; it involves strategically configuring resources (Apascaritei and Elvira, 2022), optimizing power structures (Lengnick-Hall et al., 2013), and minimizing costs (Becker and Huselid, 2010) to proactively respond to external changes. While extensive research has examined the impact of SHRM on organizational performance (Gurbuz and Mert, 2011; Harris and Ogbonna, 2001), work experiences (Becker and Huselid, 2010; Brown et al., 2009), and human capital career development (Baldi and Trigeorgis, 2020), there is a notable gap in studies positioning SHRM as a critical enabler of dynamic capabilities, particularly in fostering innovation within SMEs. This oversight is significant given the increasing complexity and uncertainty of contemporary business landscapes. Based on the view of dynamic capability, our study advances the DCV by proposing a novel ‘learning-resilience’ pathway that sequentially links SHRM practices to innovation through capability-building processes. Furthermore, we uncover the nonlinear moderating role of environmental dynamism, which challenges the conventional assumption of linear contingency relationships. This dual focus on internal capability orchestration and external environmental adaptation offers a holistic framework for understanding SME innovation in turbulent contexts.

Environmental dynamism, characterized by uncertainty, complexity, and unpredictability (Teece, 2007), challenges organizational innovation within SMEs. Existing research highlights that environmental dynamism exacerbates organizational vulnerability (Oh and Kim, 2022), impacting innovation, particularly for SMEs with limited resources (Alam et al., 2022). Despite this Fingding, there is a lack of studies comprehensively delineating how environmental dynamism shapes SMEs’ innovation pathways (Oh and Kim, 2022). Drawing from the dynamic capability perspective, our study posits that environmental dynamism influences SMEs’ innovation across two stages: “*capability utilization*” and “*capability reconfiguration*.” In the “*capability utilization*” stage, moderate environmental dynamism enhances SHRM’s positive effects on innovation by fostering knowledge acquisition, sharing, storage, and utilization, activating a learning environment (Argote et al., 2003), enhancing robustness, and sensitivity in risk response (Tierney, 2003). Conversely, in the “*capability reconfiguration*” stage, excessive dynamism restricts knowledge updating, increases the complexity and cost of learning, and diminishes response effectiveness, impairing innovation capabilities. Unlike prior studies that treat environmental dynamism as a linear moderator, our framework posits a threshold effect: moderate dynamism amplifies SHRM’s impact by stimulating learning agility, whereas excessive dynamism disrupts resource reconfiguration, thereby attenuating innovation. This dual-phase model (*capability utilization* vs. *reconfiguration*) extends the DCV by delineating how environmental thresholds shape the efficacy of a firm’s internal capabilities.

Based on dynamic capability theory, our research presents an integrated framework encapsulating both internal and external factors influencing SMEs’ innovation. This framework addresses three key questions: (1) How does SHRM impact SMEs’ innovation? (2) How do organizational learning and resilience mediate the relationship between SHRM and innovation? (3) How does environmental dynamism moderate the relationship between SHRM and SMEs’ innovation? The subsequent section delves into relevant literature, outlines study hypotheses, presents methodology, and reveals empirical results. The concluding section discusses managerial implications and future research directions.

## 2 Theoretical background and hypothesis development

### 2.1 Theoretical background: dynamic capabilities viewpoint

Dynamic capabilities refer to an organization’s proficiency in flexibly acquiring, integrating, and reorganizing its resources and skills to adapt to changing environments, which is crucial for organizational adaptability (Teece et al., 1997). This concept fundamentally includes three components: resource acquisition, integration, and reorganization (Teece et al., 1997). With the advancement of research, scholars have combined external environmental changes with internal resource allocation within organizations (Schreyögg and Kliesch-Eberl, 2007), rendering the dynamic capabilities theory more comprehensive. From this perspective, existing studies have highlighted the pivotal role of organizational learning in developing dynamic capabilities. For example, the dynamic capabilities model proposed by Pavlou and El Sawy (2006) illustrates in detail how firms can leverage learning mechanisms to bolster their dynamic capabilities through various stages. Specifically, their longitudinal study of high-tech firms demonstrates that dynamic capabilities operate through a cyclical process of “*opportunity sensing-resource reconfiguration-organizational transformation*” which in the SHRM context manifests as the tripartite interaction of cross-functional teams (resource acquisition), knowledge-sharing systems (resource integration), and agile performance evaluation (resource recombination) (Ho et al., 2023). Additionally, dynamic capabilities are pertinent to how firms optimize their strategic positioning to align with external relationships (Wang and Ahmed, 2004). Scholars have emphasized the importance of continuous resource allocation and strategic adjustments in response to external environmental changes to achieve evolutionary adaptability (Helfat et al., 2009).

Based on the viewpoint of dynamic capabilities, our study proposes a theoretical framework for the SHRM of SMEs, aimed at fostering their innovative development. It also offers a detailed analysis of the internal and external factors influencing the development of innovative capabilities in SMEs. Internally, the organizational learning ability and resilience, based on strategic human resource allocation, form the ‘*learning-resilience*’ pathway for organizational innovation in SMEs. Specifically, SHRM in SMEs plays a crucial role in promoting organizational innovation by enhancing organizational learning and resilience (Kantur and İşeri-Say, 2012). Through facilitating the processes of knowledge acquisition, dissemination, interpretation, integration, and institutionalization, SHRM cultivates a learning environment conducive to building absorptive capacity and resilience within the organization (Flores et al., 2012). This resilience enables SMEs to swiftly adapt to market changes, maintain continuous innovation, and ultimately achieve superior performance (Eisenhardt and Martin, 2000). Consequently, SHRM in SMEs significantly influences organizational learning and resilience, thereby fostering organizational innovation.

Externally, environmental dynamism, including complexity and changes, present challenges to organizational innovation activities, creating a ‘*dynamic-changing*’ environment for SMEs. Specifically, environmental dynamism exerts an inverted ‘U-shaped’ moderating effect on the relationship between SHRM and organizational innovation. Moderate environmental dynamism act as beneficial external stimuli, promoting organizational innovation. However, an excessively dynamic environment may impede innovation (Meyers, 1990). As evidenced by Jansen et al.’s (2009) study of Dutch manufacturing firms, when technological change cycles fall within the 3–5 year range (moderate dynamism), SHRM practices such as structured training systems and career development channels can maximize the efficiency of employee knowledge renewal. However, when the technological cycle shortens to 6–12 months (high dynamism), traditional HR planning inhibits rapid trial-and-error capabilities (Do et al., 2022). This threshold effect is particularly pronounced when the rate of environmental change exceeds the knowledge half-life in a given sector (Baldwin and Lin, 2002), necessitating a paradigm shift in SHRM from emphasizing job competency to building dynamic skill portfolios (Apascaritei and Elvira, 2022).

We extend current research by investigating how SHRM impacts organizational innovation in SMEs within increasingly complex environments. Specifically, the internal innovation pathway of SMEs is shaped by the ‘*learning-resilience*’ mechanisms of organizational learning and resilience, while the external environment is defined by ‘*dynamic-changing*’. These internal ‘*learning-resilience*’ forces, combined with the intensifying process of environmental dynamism, significantly influence the development of SMEs’ innovation capacity. They create a dynamic balance as environmental dynamism progresses from weak to strong. Particularly at low levels of environmental dynamism, the positive effects of strong internal organizational factors, such as learning and resilience, are magnified. During this phase, gradual environmental dynamism can enhance the positive impact of SHRM on organizational innovation. This corresponds to the ‘*capability utilization*’ stage in dynamic capabilities theory, enabling organizations to fully leverage their existing capabilities to adapt to and address external changes (Hoque et al., 2022). However, when environmental dynamism becomes excessive, external uncertainties can undermine internal robustness, causing established SHRM activities to fail and disrupting the robust internal innovation environment fostered by organizational learning and resilience (Williams et al., 2017). As a result, there is a notable decline in the organization’s innovation capability. This highlights the need for ‘*capability reconfiguration*’ within dynamic capabilities theory, necessitating organizations to reconfigure and realign their resources and capabilities in response to significant environmental changes (Hoque et al., 2022).

### 2.2 SHRM and organizational innovation in SMEs

In the context of rapidly shifting competitive dynamics and technological disruptions, SMEs increasingly prioritize cultivating innovation capacity as a strategic imperative for sustained competitiveness (Madhavan and Grover, 1998). Grounded in organizational dynamics, SHRM emerges as a critical driver of SMEs’ innovative capabilities. This study contends that SHRM practices in SMEs positively influence organizational innovation capacity, mediated through dynamic capability development (Chen and Huang, 2009).

SHRM is instrumental in creating environments that nurture innovation. Strategic practices—including staffing, training, and performance-based compensation—enable SMEs to attract talent with diverse expertise and innovative orientations (Chen and Huang, 2009). Such practices not only elevate employee engagement but also institutionalize a culture of iterative learning and experimentation, prerequisites for innovation (Damanpour, 1991). For example, selective recruitment targeting individuals with creative problem-solving abilities establishes a foundation for disruptive ideation (Atuahene-Gima, 1996).

SHRM enhances SMEs’ dynamic capabilities—the capacity to adapt resources to volatile markets (Teece et al., 1997). Recent studies have demonstrated that flexibility-oriented HRM systems, characterized by cross-functional staffing and adaptive performance management, are particularly effective in fostering innovation resilience during crises (Roumpi, 2023). By fostering absorptive capacity (i.e., assimilating and applying external knowledge) through tailored training programs, SMEs equip employees to transform insights into actionable innovations (Cohen and Levinthal, 1990). Participatory decision-making and appraisal systems that reward creativity further reinforce employees’ ownership of innovation outcomes (Jiménez-Jiménez and Sanz Valle, 2005).

SHRM facilitates knowledge integration critical to innovation. Tacit knowledge, often embedded in human capital, drives novel solutions when shared across teams (Nonaka, 2009). HR practices promoting collaboration and knowledge exchange—such as cross-functional projects—enable SMEs to synthesize disparate ideas into market-ready innovations (Chen and Huang, 2009). Incentive structures aligned with innovation metrics further motivate risk-taking, amplifying organizational agility (Scarbrough, 2003). Consequently, we hypothesize that:

Hypothesis 1: SHRM in SMEs relates positively to their organizational innovation.

### 2.3 The mediating role of organizational learning

The relationship between SHRM and organizational innovation in SMEs has gained substantial attention in recent years, as these firms endeavor to maintain competitiveness in rapidly changing markets. Grounded in the dynamic capabilities framework, which highlights the significance of organizational processes in reshaping and redeploying internal and external competencies to respond to shifting market demands (Teece et al., 1997), this research suggests that SHRM’s positive impact on organizational innovation in SMEs is mediated by their organizational learning capability.

SHRM includes various practices such as recruitment, training, employee participation, performance appraisal, and compensation management, all of which have been shown to cultivate an environment conducive to learning and innovation (Chen and Huang, 2009). These practices not only attract and retain talented employees but also enhance their commitment and motivation, fostering a culture of continuous learning and adaptation. Specifically, training programs facilitate the acquisition of new knowledge and skills, while performance appraisal and compensation systems incentivize innovative behaviors and outcomes.

The institutionalization of learning processes through SHRM creates social capital that binds human resources into collaborative networks, enabling efficient knowledge recombination (Ben-Hador and Yitshaki, 2025). Organizational learning capability, defined as the firm’s ability to create, retain, and transfer knowledge to improve performance (Argote et al., 2003), is crucial in mediating the SHRM-organizational innovation relationship. This capability is exhibited through five interconnected subprocesses: information acquisition, information distribution, information interpretation, knowledge integration, and organizational memory (Flores et al., 2012). SMEs that adeptly manage these subprocesses are better positioned to identify and seize new opportunities, adapt to market shifts, and develop innovative products and services.

From a dynamic capabilities perspective, information acquisition allows SMEs to collect relevant data and insights from both internal and external sources, forming the basis for innovation (Brunswicker and Vanhaverbeke, 2015). Information distribution ensures this knowledge is disseminated throughout the organization (Bergquist et al., 2001), fostering a collective understanding and shared language for innovation. Information interpretation entails making sense of the acquired data, generating new insights and ideas (Choo, 1996). Knowledge integration aligns these insights with the firm’s existing competencies, facilitating the development of novel solutions (Salunke et al., 2019). Lastly, organizational memory enables the firm to retain and leverage past learning experiences, thereby accelerating the innovation process (Akgün et al., 2012). Given the pivotal role of organizational learning capability in transforming SHRM practices into organizational innovation, we hypothesize that:

Hypothesis 2: Organizational learning mediates the positive relationship between SHRM and organizational innovation in SMEs.

### 2.4 The mediating role of organizational resilience

SHRM includes practices such as staffing, training, employee involvement, performance appraisal, and compensation management, which have been shown to be essential in building organizational resilience (Chen and Huang, 2009). This study, grounded in the dynamic capabilities perspective, suggests that organizational resilience acts as a crucial mediating role between SHRM practices and organizational innovation in SMEs.

SHRM practices like strategic staffing that focus on selecting individuals with creative abilities and innovative traits contribute to developing a workforce capable of adapting to changes and generating new ideas (Chen and Huang, 2009). Training programs designed to enhance employees’ knowledge and skills further equip them for innovative tasks (Hou et al., 2017). Additionally, employee involvement in decision-making processes and performance appraisals that recognize and reward innovative behaviors cultivate a culture of creativity and risk-taking (Chen and Huang, 2009). These SHRM practices collectively enhance SMEs’ organizational resilience by creating a strong and adaptable workforce capable of withstanding and recovering from external shocks (Kantur and İşeri-Say, 2012). Organizational resilience, in turn, significantly facilitates organizational innovation.

Recent evidence from technology SMEs confirms that strategic HRM configurations emphasizing ambidextrous capabilities—simultaneous exploitation of existing competencies and exploration of new practices—significantly enhance adaptive resilience (Ho et al., 2023). From the dynamic capabilities viewpoint, organizational resilience includes three key dimensions: robustness, sensitivity, and integrity (Tierney, 2003; Kantur and İşeri-Say, 2012). Robustness is the ability of an organization to endure and recover from disruptions without substantial loss of functionality (Tierney, 2003). This quality helps SMEs maintain stability during crises, thus fostering a conducive environment for ongoing innovation. Sensitivity involves an organization’s ability to quickly detect and respond to external changes (Kantur and İşeri-Say, 2012). This rapid adaptation enables SMEs to seize emerging opportunities and integrate new knowledge into their operations, boosting their innovative capabilities. Lastly, integrity refers to the availability and sufficiency of resources necessary for organizational functioning and adaptation (Tierney, 2003). A resilient organization with a comprehensive and rich resource base is better positioned to support and sustain innovative activities over time. By fostering a robust, responsive, and resourceful organization, SHRM practices enable SMEs to effectively manage and leverage their human capital in the pursuit of innovative strategies and solutions. Consequently, we hypothesize that:

Hypothesis 3: Organizational resilience mediates the positive relationship between SHRM and organizational innovation in SMEs.

### 2.5 The “learning-resilience” innovation path for SMEs based on organizational learning and organizational resilience

We further investigate the relationship between organizational learning and organizational resilience. The subprocesses of organizational learning collectively enhance an organization’s capacity to detect and interpret environmental changes, integrate new knowledge, and embed it within organizational memory, thus fostering resilience (Eisenhardt and Martin, 2000; Teece et al., 1997).

Information acquisition, as the initial phase of organizational learning, equips SMEs with the insights needed to anticipate and respond to market shifts. This proactive approach corresponds to the robustness aspect of organizational resilience, enabling firms to withstand external shocks with minimal disruption (Weick and Sutcliffe, 2001). Conversely, information distribution ensures that knowledge is widely disseminated within the organization, promoting a culture of collaboration and collective problem-solving, which is crucial for swift responsiveness and adaptability, reflecting the sensitivity aspect of resilience (Argote et al., 2003). Information interpretation, knowledge integration, and organizational memory collectively play a critical role in enhancing the integrity of an organization’s resource base. By interpreting new information and integrating it with existing knowledge, firms can devise innovative solutions and strategies that strengthen their adaptive capacity (Nonaka, 2009). Organizational memory, serving as a repository of past experiences and lessons, provides a foundation for future decision-making, further enhancing resilience by preventing the recurrence of past mistakes (Levitt and March, 1988).

Based on Hypotheses 2 and 3, we infer that organizational learning and resilience act as chain mediators in the process by which SMEs’ SHRM practices positively relate to organizational innovation. From the perspective of dynamic capabilities, organizational learning derived from the development of SHRM practices enables firms, particularly SMEs, to boost their resilience and subsequently enhance their innovative capacities.

Hypothesis 4: Organizational learning and organizational resilience serve as chain mediators in the process by which SMEs' SHRM relates positively to their organizational innovation. Specifically, SHRM in SMEs relates positively to organizational learning, which then boosts organizational resilience. This, in turn, has a positive effect on organizational innovation.

### 2.6 The “dynamic-changing” environment for organizational innovation in SMEs: the moderating role of environmental dynamism

In today’s highly volatile and unpredictable business environment, organizational innovation is critical for SMEs to navigate challenges posed by environmental dynamism (Eisenhardt and Martin, 2000). Based on the dynamic capability theory, the efficacy of SHRM in promoting organizational innovation is closely tied to its adaptability to external environmental changes (Teece et al., 1997). This emphasizes the need to investigate how environmental dynamism moderates the relationship between SHRM and organizational innovation.

SHRM includes practices such as recruitment, training, employee involvement, performance appraisal, and compensation management, all shown to positively impact organizational innovation (Chen and Huang, 2009). In contexts of low environmental dynamism, SMEs face fewer rapid and unpredictable market shifts. Under these conditions, SHRM practices can be more effectively implemented, enhancing organizational learning, resilience, and ultimately, innovation (Do et al., 2022). Specifically, low environmental dynamism allows strategic recruitment to attract individuals with the necessary knowledge and skills, fostering a workforce conducive to innovation (Brockbank, 1999). Training programs, designed to update employees with the latest knowledge and techniques, can be systematically executed, thus enhancing their innovative capabilities (Jaw and Liu, 2003). Furthermore, employee involvement in decision-making fosters a culture of creativity and openness to new ideas, crucial for innovation (Damanpour, 1991). Performance appraisals and compensation systems aligned with innovation outcomes provide clear incentives for employees to engage in innovative activities (Hurley and Hult, 1998).

Enhanced organizational learning through these SHRM practices, moderated by low environmental dynamism, strengthens organizational resilience (Lengnick-Hall et al., 2011). This resilience enables firms to adapt efficiently to external changes, seize opportunities, and develop innovative solutions (Kim and Mauborgne, 1999). Organizations with stronger resilience are better positioned to innovate, as resilience provides a foundation for experimenting, learning, and innovating in the face of challenges (Richtér and Löfsten, 2014). Consequently, we hypothesize that:

Hypothesis 5: Environmental dynamism moderates the relationship between SHRM and organizational innovation. Specifically, the positive relationship between SHRM and organizational innovation is improved when environmental dynamism is low.

Based on the dynamic capability view, we continue posit that high environmental dynamism weakens and may even negate the positive relationship between SHRM and organizational innovation. SHRM includes a comprehensive approach involving recruitment, training, participation, performance appraisal, and compensation management (Chen and Huang, 2009). These elements collectively build an organizational capacity for innovation by fostering an environment that promotes creativity and adaptability among employees. However, in increasingly volatile and unpredictable external environments, the efficacy of these SHRM practices in driving organizational learning may be undermined.

From a dynamic capability perspective, organizations need dynamic capabilities to reconfigure resources and routines in response to changing market conditions (Teece et al., 1997). Environmental dynamism, marked by rapid technological changes, market fluctuations, and heightened competition, demands higher levels of organizational agility and flexibility (Eisenhardt and Martin, 2000). Under such conditions, traditional SHRM practices that focus on stability and routine may no longer be adequate and may even impede the organization’s ability to adapt quickly and innovate effectively. For example, recruitment processes that prioritize expertise and experience over adaptability and learning agility may fail to attract individuals capable of thriving in a rapidly changing environment (Ratten, 2020). Similarly, training programs centered on established practices and procedures might limit employees’ ability to acquire new skills and knowledge essential for innovation in dynamic contexts (Sanders et al., 2021). Additionally, high levels of participation and involvement in decision-making processes can become cumbersome and inefficient in fast-paced environments, potentially slowing the organization’s response time (Shipton et al., 2017). Moreover, performance appraisal systems that reward consistency and reliability might discourage risk-taking and experimentation, both crucial for innovation (Do et al., 2022). Finally, compensation structures misaligned with the dynamic market demands may fail to incentivize employees to engage in innovative behaviors (Jiang et al., 2021).

Drawing from Do et al. (2022), we argue that under high environmental dynamism, the positive relationship between SHRM and organizational learning is attenuated, weakening organizational resilience, which is critical for sustaining innovation efforts (Lengnick-Hall et al., 2011). This nonlinear pattern aligns with the dynamic capability reconfiguration threshold proposed by Ho et al. (2023), where moderate environmental shifts stimulate capability utilization, whereas excessive turbulence demands radical HRM restructuring. SHRM practices that prove effective in stable contexts may become misaligned when the velocity of environmental change exceeds the organizational learning cycles (Saha et al., 2016). Organizational resilience, defined as the ability to absorb shocks, adapt, and transform in response to external disruptions, is vital for innovation (Coutu, 2002). When SHRM practices are not aligned with dynamic environmental demands, they may hinder resilience development, thus impeding the organization’s ability to innovate effectively. We hypothesize that:

Hypothesis 6: Environmental dynamism moderates the positive relationship between SHRM and organizational innovation. Specifically, when environmental dynamism is high, the positive relationship between SHRM and organizational innovation is weakened and even reduced.

**Figure 1:**
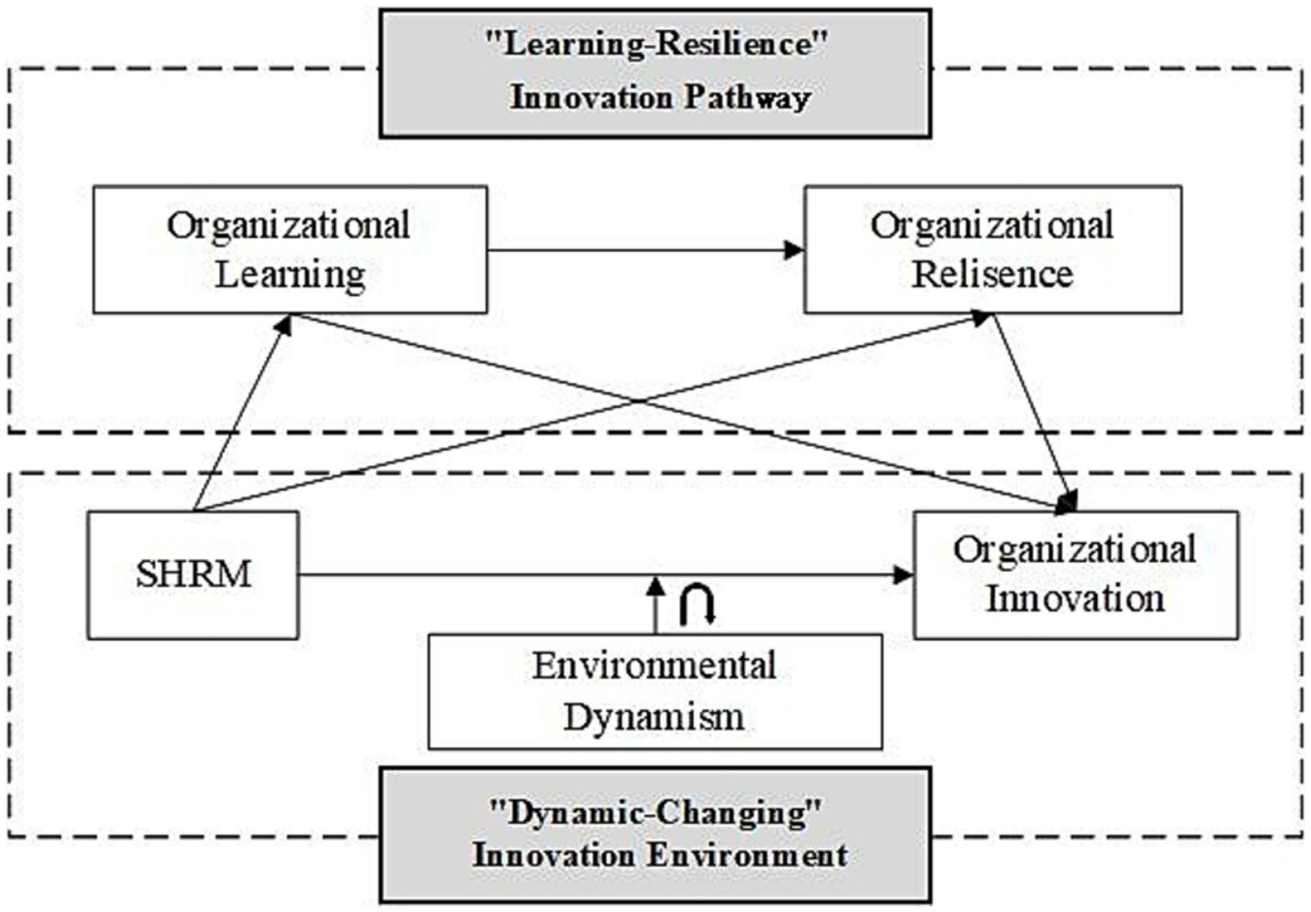
Theoretical framework.

Based on the above analysis, our research framework model is derived (Figure 1).

## 3 Research methodology

### 3.1 Data collection and sample

A multistage sampling approach was adopted to ensure the representativeness of SMEs in China’s Yangtze River Delta region (e.g., Shanghai and Suzhou). As the most economically dynamic area, contributing 24% of China’s GDP (4.54 trillion US dollars; Li et al., 2024), this region hosts over 3 million SMEs with distinctive innovation ecosystems. We specifically targeted technology-intensive industries (software development, high-tech services, information transmission, and biomedicine) as they constitute 68% of the registered SMEs in regional industrial parks, which aligns with our research focus on innovation-driven companies. The three-wave survey (August–October 2023) employed stratified random sampling across four provinces (Shanghai 32%, Jiangsu 28%, Zhejiang 25%, and Anhui 15%) proportional to their SME distributions. From the official SME registry, 500 eligible firms (50–500 employees, established 3–15 years) were randomly selected for this study. Through collaborative partnerships with local chambers of commerce and innovation incubators, we obtained participation commitments from 434 enterprises (86.80% response rate).

Before commencing our research, we conducted offline field visits and online email consultations to thoroughly explain the academic goals of our survey, the confidentiality of individual responses, and the need for multiple rounds of questionnaires to our participants. Once their consent was obtained, we distributed the questionnaires through offline mail and online email, instructing participants to complete and return them within two weeks (questionnaires submitted after the deadline were deemed invalid). The first phase collected participants’ personal demographic information (as control variables) and measurement items for the SHRM variable. Participants were asked to provide the initial letters of their names and the last four digits of their phone numbers to facilitate subsequent matching of questionnaire data. One month after the first phase was completed, we distributed the second-phase questionnaires, focusing on the variables of organizational learning, organizational resilience, and environmental dynamism. A month after the second-phase survey, we initiated the third-phase data collection, which primarily targeted the measurement of the organizational innovation variable. After meticulous screening, 256 valid questionnaires were identified, translating to an effective response rate of 59.26%. The final 256 valid responses showed industry distribution consistency with regional tech SME composition (*χ*^2^ = 1.32, *p* = 0.72), confirming sample representativeness. Post-hoc power analysis (G*Power 3.1) indicated 89% power to detect medium effects (*f* ^2^ = 0.15), exceeding the conventional thresholds.

The sample demographics yield interesting insights (Table 1). In terms of gender, the sample is mainly male, with 145 male participants accounting for 56.6% of the total. Regarding age, the largest group lies within the 26–30 age range, constituting 37.9% of the sample with 97 participants. Position-wise, 114 respondents (44.5%) have a management background, highlighting the significance of managerial perspectives in this study. Moreover, the majority of respondents (76.5%) are employed in businesses established within the past five years, indicating a focus on emerging enterprises (SMEs). The industry sectors represented are mainly software development, high-tech services, and information transmission, collectively accounting for 76.5% of the total sample. This alignment with technology-driven sectors emphasizes the relevance of the study in the contemporary business context. Regarding enterprise ownership, private enterprises make up the largest proportion, accounting for 46.9% of the total. In terms of firm size, enterprises with fewer than 50 employees are most common, constituting 32% of the total sample, with 82 enterprises falling into this category.

**Table 1:** Demographic information (*N* = 256).

Characteristics	Classifications	Frequency	Percentage
Gender	Male	145	56.6
	Female	111	43.4
Age	Below 25	11	4.3
	26–30	97	37.9
	31–40	71	27.7
	41–50	52	20.3
	Above 51	25	9.8
Position	Grass-roots employees	142	55.5
	Grass-roots managers	97	37.9
	Middle and senior managers	17	6.6
Firm ages (years of establishment)	Below 1	23	9
	1–3	81	31.6
	3–5	92	35.9
	5–10	40	15.6
	Above 10	20	7.8
Industry type	Electronic information	34	13.3
	Biomedical medicine	26	10.2
	Software development	59	23
	High-tech services	74	28.9
	Information transmission	63	24.6
Firm ownership	Private enterprises	120	46.9
	Joint enterprises	29	11.3
	Public enterprises	64	25
	Foreign capital enterprises	43	16.8
Firm size (no. of employees)	0–50	82	32
	51 ~ 200	72	28.1
	201–500	66	25.8
	Above 500	36	14.1

### 3.2 Measures

To address the research hypotheses, a comprehensive measurement framework was developed using a 7-point Likert scale ranging from 1 (“strongly disagree”) to 7 (“strongly agree”). This methodology enabled the assessment of SHRM, organizational learning, resilience, innovation, and environmental dynamics.

#### 3.2.1 SHRM

Based on the work of Chen and Huang (2009), the SHRM scale includes five dimensions: recruitment, employee training, compensation, performance appraisal, and employee engagement. This 16-item scale captures the multifaceted nature of SHRM practices.

#### 3.2.2 Organizational learning

The measurement of organizational learning was derived from the scale by Flores et al. (2012), which includes five dimensions: information acquisition, distribution, interpretation, integration, and learning memory. This scale consists of 23 items.

#### 3.2.3 Organizational resilience

For assessing organizational resilience, the scale developed by Lengnick-Hall and Beck (2003) was employed, dividing resilience into three dimensions: robustness, sensitivity, and integrity, across 9 items.

#### 3.2.4 Organizational innovation

Drawing from the research by Chen and Huang (2009) and Ibarra (1993), organizational innovation was measured across administrative and technological dimensions using a 7-item scale.

#### 3.2.5 Environmental dynamism

The measurement of environmental dynamism was based on the work of Jaworski and Kohli (1993) and other researchers. This 4-item scale assesses the degree of rapid change and uncertainty in the environment.

Additionally, control variables such as firm age, industry type, firm ownership, and firm size were included to account for contextual influences, drawing from the insights of Govindarajan and Kopalle (2006).

## 4 Analysis and results

### 4.1 Reliability and validity

Using SPSS 26.0, we conducted a thorough analysis to assess the *reliability* and *validity* of the measurement scales used in this study. As shown in Table 2, the Cronbach’s alpha coefficients for all variables exceeded the recommended threshold of 0.70 by Hair et al. (1998), indicating high internal consistency and reliability. This suggests that the items in the scales consistently and stably measured their respective variables with minimal measurement errors, thus effectively reflecting the underlying constructs (Andrews and Kacmar, 2001).

**Table 2:** Reliability and validity of variables.

Variables	Items	Cronbach’s Alpha	Item count
SHRM		0.929	16
	Recruitment	0.880	3
	Training	0.860	4
	Compensation	0.852	3
	Performance evaluation	0.777	3
	Employee engagement	0.843	3
Organizational learning		0.946	23
	Information acquisition	0.891	4
	Information distribution	0.896	4
	Information interpretation	0.902	4
	Information integration	0.919	5
	Learning memory	0.924	6
Environmental dynamism		0.932	4
Organizational resilience		0.908	9
	Robustness	0.903	4
	Sensitivity	0.873	3
	Integrity	0.830	2
Organizational innovation		0.889	7
	Administrative innovation	0.884	4
	Technological innovation	0.875	3

To further verify the strong construct and discriminant validity among the primary variables, we utilized Mplus 8.3 to perform a *confirmatory factor analysis (CFA)* on five variables: SHRM, organizational learning, organizational resilience, organizational innovation, and environmental Dynamism. The results indicated that the five-factor model demonstrated an excellent fit to the data, with fit indices of *χ*^2^/df = 1.927, RMSEA = 0.060, SRMR = 0.047, CFI = 0.949, and TLI = 0.939. Compared to alternative models, the fit indices of the five-factor model were superior, as shown in Table 3. These results underscore the robust structural and discriminant validity of the variables (Argote et al., 2003).

**Table 3:** Confirmatory factor analysis.

Model	$x^{2}$	*df*	$x^{2}$ /*df*	△ $x^{2}$ (*df*)	*CFI*	*TLI*	*RMSEA*	*SRMR*
Standard criteria			<3		>0.9	>0.9	<0.08	<0.08
Five-factor model X; M1; M2; Y; W	273.606	142	1.927		0.949	0.939	0.060	0.047
Four-factor model X + M1; M2; Y; W	585.161	146	4.008	311.555(4)	0.831	0.802	0.108	0.081
Three-factor model X + M1 + M2; Y; W	761.995	149	5.114	488.389(7)	0.764	0.730	0.127	0.092
Two-factor model X + M1 + M2 + Y; W	808.207	151	5.352	534.601 (9)	0.747	0.714	0.130	0.095
One-factor model X + M1 + M2 + Y + W	1384.722	152	9.110	1111.116 (10)	0.526	0.467	0.178	0.128
Five-factor model + CMV X; M1; M2; Y; W; CMV	195.675	123	1.591	77.931 (19)	0.972	0.961	0.048	0.038

X = SHRM; M1 = Organizational Learning; M2 = Organizational Resilience; Y=Organizational Innovation; W = Environmental Dynamism; “+”indicates two factors combined into one; CMV refers for common method variance; all Δ significant at *p* < 0.001.

### 4.2 Common method bias

To address concerns about common method bias, which could potentially affect the study’s model construction, we adopted two approaches. Firstly, the *Harman’s single-factor test* was conducted. Without factor rotation, the first common factor accounted for 29.095% of the variance, below the accepted threshold of 40% (Podsakoff et al., 2003). This initial result suggested that no single factor dominated, indicating an insignificant impact of common method bias on the study’s model. Additionally, a *common method factor (CMV)* was introduced into the five-factor model, and a comparative analysis of model fit was performed. The inclusion of the CMV did not significantly improve the model fit (Table 3), with fit indices of *χ*^2^/df = 1.591, RMSEA = 0.048, SRMR = 0.038, CFI = 0.972, and TLI = 0.961. This further validated that common method bias did not significantly influence the model construction (Hamilton and Nickerson, 2003).

### 4.3 Correlation analysis

Table 4 illustrates the correlations derived from our empirical study. The results reveal that SHRM practices have a significant positive correlation with organizational learning, environmental dynamism, organizational resilience, and ultimately, organizational innovation (*r* = 0.408, 0.426, 0.380, 0.414, respectively; all *p* < 0.01). These findings suggest that effective SHRM practices create a conducive environment for the acquisition, sharing, and application of knowledge within organizations, thereby enhancing learning capabilities. Additionally, environmental dynamism, characterized by rapid changes and uncertainties in the external operating context, exhibit a significant positive relationship with organizational learning (*r* = 0.261, *p* < 0.01), supporting previous studies that emphasize the need for adaptability and flexibility in turbulent environments (Dyer and Nobeoka, 2000; Hansen, 1999). Similarly, organizational resilience, defined as the ability to recover and adapt in the face of adversities, shows a strong positive correlation with both organizational learning (*r* = 0.363, *p* < 0.01) and organizational innovation (*r* = 0.509, *p* < 0.01). This underscores the crucial role of resilient organizations in leveraging learning experiences to foster innovation. Collectively, these correlations provide preliminary empirical support for our subsequent hypothesis testing, highlighting the interconnectedness among SHRM practices, organizational innovation, and various organizational outcomes.

**Table 4:** Correlation analysis.

	1	2	3	5	6	7	8	9	10	11	12
1. SHRM	1.000										
2. Organizational learning	0.408^**^	1.000									
3. Environmental dynamism	0.426^**^	0.261^**^	1.000								
4. Organizational resilience	0.380^**^	0.363^**^	0.283^**^								
5. Organizational innovation	0.414^**^	0.509^**^	0.490^**^	1.000							
6. Gender	−0.036	0.070	0.017	0.072	1.000						
7. Age	0.063	0.060	0.106	0.096	0.054	1.000					
8. Position	0.016	0.021	0.119	0.154^*^	0.143^*^	0.276^**^	1.000				
9. Firm ages	0.079	0.059	0.159^*^	0.128^*^	−0.012	0.059	0.012	1.000			
10. Industry type	0.093	0.029	−0.069	0.020	−0.006	0.097	−0.294^**^	−0.013	1.000		
11. Firm ownership	0.090	0.098	0.059	0.039	−0.020	0.087	−0.023	0.292^**^	0.065	1.000	
12. Firm size	−0.071	0.067	−0.089	−0.011	−0.040	0.010	−0.034	0.029	0.022	0.142^*^	1.000
*M*	4.599	4.662	4.505	4.782	0.434	2.934	1.512	2.816	3.414	2.117	2.219
*SD*	1.061	1.039	1.639	1.229	0.497	1.070	0.620	1.056	1.320	1.176	1.047

*p < 0.05, **p < 0.01.

### 4.4 Hypothesis testing

Table 5 presents the results of the regression analysis, with *Model 1c* serving as the baseline model that incorporates control variables for organizational innovation. *Model 2c*, building on *Model 1c*, includes SHRM practices. The regression results indicate a positive and significant effect of SHRM on organizational innovation (*b* = 0.473, *p* < 0.001), supporting Hypothesis 1. To further clarify the mediating mechanism of organizational learning, we initially examined the positive impact of SHRM on organizational learning as shown in *Model 2b* in Table 5 (*b* = 0.414, *p* < 0.001). This linkage underscores how SHRM institutionalizes sustainable learning practices through three mechanisms: (1) systematic training programs that update employees’knowledge repositories (Cohen and Levinthal, 1990), (2) cross-functional collaboration fostering knowledge codification (Nonaka, 2009), and (3) ambidextrous reward systems balancing exploratory and exploitative learning (Jansen et al., 2009). Subsequently, *Model 3c* was utilized to assess the influence of organizational learning on innovation within SMEs (*b* = 0.466, *p* < 0.001). These findings suggest that SHRM positively affects organizational innovation in SMEs through a sustainable learning-resilience loop in which knowledge acquisition is embedded in organizational routines (Argote et al., 2003). To verify the mediating role of organizational learning, we conducted a mediation effect test using the Bootstrap plugin in Table 6. The results indicate that the direct effect of SHRM on organizational innovation was reduced (from 0.182 to 0.158) but remained significant (95% CI = [0.090, 0.235], excluding 0), confirming organizational learning as a partial mediator that transforms human capital investments into reusable knowledge assets, thereby supporting Hypothesis 2.

**Table 5:** Results of regression analysis of organizational innovation.

Variable	Organizational learning	Organizational learning	Organizational resilience	Organizational resilience	Organizational resilience	Organizational innovation	Organizational innovation	Organizational innovation	Organizational innovation
	Model 1a	Model 2b	Model 1b	Model 2b	Model 3b	Model 1c	Model 2c	Model 3c	Model 4c
Intercept	3.988^***^	2.153^***^	3.933^***^	2.144^***^	1.597^***^	3.575^***^	1.476^**^	0.474	−0.057
Gender	0.174	0.210	0.161	0.196	0.143	0.158	0.200	0.102	0.054
Age	0.034	0.024	0.057	0.047	0.041	0.032	0.021	0.009	−0.004
Position	0.041	0.022	0.002	−0.016	−0.021	0.320^*^	0.299^*^	0.288^*^	0.295^**^
Firm ages	0.037	0.011	0.129	0.103	0.100	0.154^*^	0.124	0.119	0.085
Industry type A	0.454	0.530^*^	0.045	0.119	−0.016	0.408	0.495	0.248	0.254
Industry type B	0.010	0.049	−0.079	−0.041	−0.054	−0.066	−0.022	−0.045	−0.027
Industry type C	0.125	0.172	−0.097	−0.051	−0.095	0.106	0.159	0.079	0.11
Industry type D	0.223	0.105	0.158	0.043	0.017	0.366	0.231	0.182	0.177
Firm ownership A	0.097	0.094	0.255	0.253	0.229	0.058	0.056	0.012	−0.064
Firm ownership B	0.154	0.120	0.343	0.31	0.279	−0.014	−0.052	−0.109	−0.201
Firm ownership C	0.176	0.088	0.391	0.305	0.283	−0.006	−0.107	−0.148	−0.242
Firm size	0.051	0.083	0.020	0.050	0.029	−0.009	0.027	−0.012	−0.022
SHRM		0.414^***^		0.403^***^	0.298^***^		0.473^***^	0.281^***^	0.182^**^
Organizational Learning					0.254^***^			0.466^***^	0.381^***^
Organizational Resilience									0.332^***^
*R* ^2^	0.038	0.208	0.065	0.192	0.233	0.066	0.225	0.348	0.424
Δ*R*^2^	0.038	0.169^***^	0.065	0.127^***^	0.040^***^	0.066	0.159^***^	0.123^***^	0.077^***^
*F*	0.805	4.881^***^	1.418	4.432^***^	5.217^***^	1.431	5.394^***^	9.169^***^	11.798^***^

*N* = 256, with two-tailed hypothesis testing and unstandardized regression coefficients reported. Significance levels are denoted as *p < 0.05, **p < 0.01, and ***p < 0.001. Dummy variables are operationalized as follows. SHRM: Strategic Human Resource Management. Industry type: Biomedical medicine (1 = yes, 0 = others), Software development (1 = yes, 0 = others), High-tech services (1 = yes, 0 = others), Information transmission (1 = yes, 0 = others). Firm ownership: Joint enterprises (1 = yes, 0 = others), Public enterprises (1 = yes, 0 = others), Foreign capital enterprises (1 = yes, 0 = others).

**Table 6:** Bootstrap test results of mediating effect.

Path	Effect value	S. E.	95% confidence interval		Effect ratio
			Lower	Upper	
Total indirect effect	0.473	0.067	0.341	0.606	100%
Direct effect	0.182	0.066	0.051	0.312	38.48%
Total mediating effect	0.292	0.047	0.200	0.386	61.52%
H2: SHRM→OL → OI	0.158	0.037	0.090	0.235	33.19%
H3: SHRM→OR→OI	0.099	0.031	0.043	0.166	20.93%
H4: SHRM→OL → OR→OI	0.035	0.015	0.011	0.069	7.40%

SHRM: Strategic Human Resource Management; OL: Organizational Learning; OR: Organizational Resilience; OI: Organizational Innovation.

Furthermore, we validated the mediating role of organizational resilience as an adaptive capacity to reconfigure resources (Teece et al., 1997) in the process by which SHRM affects organizational innovation capabilities in SMEs. *Model 2b* in Table 5 demonstrates the positive impact of SHRM on organizational resilience (*b* = 0.403, *p* < 0.001), highlighting that strategic staffing and flexible job designs cultivate workforce agility to absorb environmental shocks (Lengnick-Hall et al., 2011), while *Model 4c* shows the positive influence of organizational resilience on organizational innovation in SMEs (b = 0.332, *p* < 0.001). Based on these regression outcomes, SHRM positively influences organizational innovation through resilience-building practices that convert learning outcomes into risk-mitigation capabilities. To further substantiate the mediating role of organizational resilience, we employed the Bootstrap plugin for mediation effect testing in Table 6. The results showed that, compared to the direct impact of SHRM on organizational innovation, the effect value was reduced but remained significant (from 0.182 to 0.099, 95% CI = [0.043, 0.166], excluding 0) after including organizational resilience. This indicates that organizational resilience partially mediates the relationship between SHRM and innovation outputs, confirming Hypothesis 3.

Drawing on the dynamic capability view (Helfat et al., 2009), we confirmed the chain mediation effect as a synergistic process: SHRM fosters organizational learning (*Model 2b*: *b* = 0.414, *p* < 0.001), which in turn enhances organizational resilience (*Model 3b*: *b* = 0.254, *p* < 0.001), ultimately driving innovation (*Model 4c*: *b* = 0.332, *p* < 0.001). This sequential mechanism reveals that sustainable organizational effectiveness emerges from SHRM’s dual focus on knowledge creation (learning) and adaptive resource orchestration (resilience). Additionally, we conducted a chain mediation effect test using the Bootstrap plugin in Table 6. Compared to the direct impact of SHRM on organizational innovation, the effect value decreased but remained significant (from 0.182 to 0.035, 95% CI = [0.011, 0.069], excluding 0) after incorporating organizational learning and organizational resilience. This “learning-resilience” pathway demonstrates that SMEs achieve innovation sustainability not merely through isolated learning efforts but via systemic capability-building that aligns knowledge stocks with dynamic environmental demands (Zahoor et al., 2020). By validating Hypothesis 4, this finding advances the theoretical integration of SHRM and dynamic capabilities.

**Figure 2:**
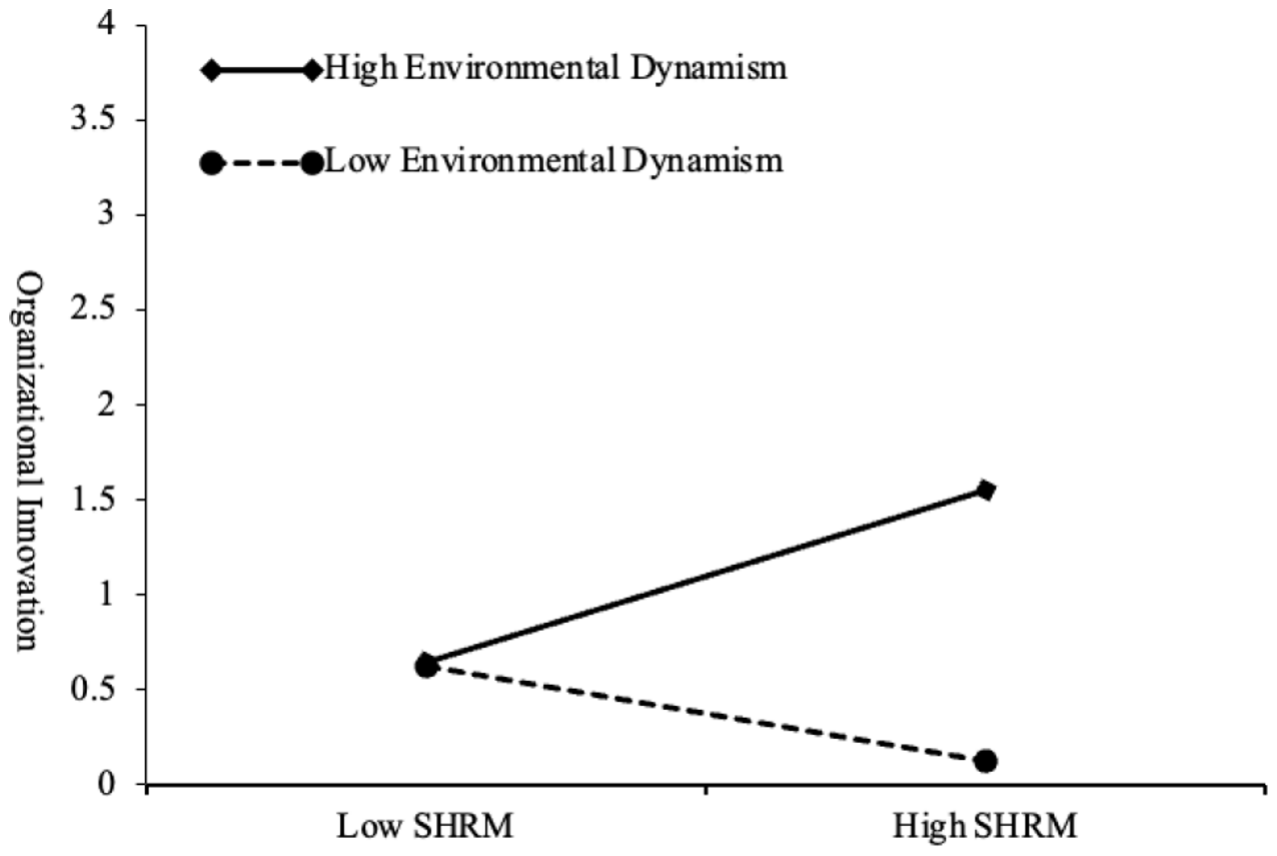
Moderating role of environmental dynamism in the relationship between SHRM and organizational innovation.

Moreover, environmental dynamism moderates the relationship between SHRM and organizational innovation in SMEs. As indicated in *Model 6c* of Table 7, the interaction between SHRM and environmental dynamism significantly enhances organizational innovation (*b* = 0.243, *p* < 0.001). This finding suggests that a suitable level of environmental dynamism amplifies the positive effects of SHRM on organizational innovation (Figure 2). It promotes organizational resilience by fostering learning and knowledge management in an appropriately dynamic environment, thereby driving organizational innovation and growth (Chen and Huang, 2009). Table 8 illustrates that when environmental dynamism is at a moderate high level (M + 1SD), SHRM has a significant positive impact on organizational innovation in SMEs (*γ* = 0.431, 95% CI [0.269, 0.593]). When environmental dynamism is at a moderate low level (M - 1SD), this effect is also significant (γ = −0.235, 95% CI [−0.383, −0.087]). This result further supports Hypothesis 5 of our study.

**Table 7:** Results of moderating role of environmental dynamism.

Variables	Organizational innovation		
	Model 5c	Model 6c	Model 7c
Intercept	1.402^***^	0.888^*^	1.032^**^
Gender	0.015	0.034	0.03
Age	−0.048	−0.058	−0.054
Position	0.243^*^	0.228^**^	0.209^*^
Firm ages	0.06	0.055	0.052
Industry type A	0.247	0.255	0.200
Industry type B	0.002	0.032	0.005
Industry type C	0.178	0.202	0.187
Industry type D	0.227	0.299	0.252
Firm ownership A	−0.091	−0.074	−0.075
Firm ownership B	−0.122	−0.065	−0.081
Firm ownership C	−0.105	−0.059	−0.041
SHRM	0.077	0.128^*^	0.323^***^
Organizational learning	0.372^***^	0.424^***^	0.420^***^
Organizational resilience	0.299^***^	0.377^***^	0.372^***^
Environmental dynamism	0.131^**^	0.097^**^	0.101^**^
Environmental dynamism^2^		−0.160^***^	−0.168^***^
SHRM*environmental dynamism		0.243^***^	0.182^***^
SHRM*environmental dynamism^2^			−0.062^***^
*R^2^*	0.55	0.661	0.678
Δ*R*^2^	0.126^***^	0.111^***^	0.018^***^
*F*	17.116^***^	25.625^***^	26.200^***^

*N* = 256, with two-tailed hypothesis testing and unstandardized regression coefficients reported. Significance levels are denoted as *p < 0.05, **p < 0.01, and ***p < 0.001. Dummy variables are operationalized as follows. SHRM: Strategic Human Resource Management. Industry type: Biomedical medicine (1 = yes, 0 = others), Software development (1 = yes, 0 = others), High-tech services (1 = yes, 0 = others), Information transmission (1 = yes, 0 = others). Firm ownership: Joint enterprises (1 = yes, 0 = others), Public enterprises (1 = yes, 0 = others), Foreign capital enterprises (1 = yes, 0 = others).

**Table 8:** Moderating role of environmental dynamism in the relationship between SHRM and organizational innovation.

	Environmental dynamism	Effect value	S. E.	95% confidence interval	
				Lower	Upper
	M-1SD	−0.235	0.075	−0.383	−0.087
SHRM→OI	M	0.098	0.061	−0.022	0.218
	M + 1SD	0.431	0.082	0.269	0.593

SHRM: Strategic Human Resource Management; OI: Organizational Innovation.

**Figure 3:**
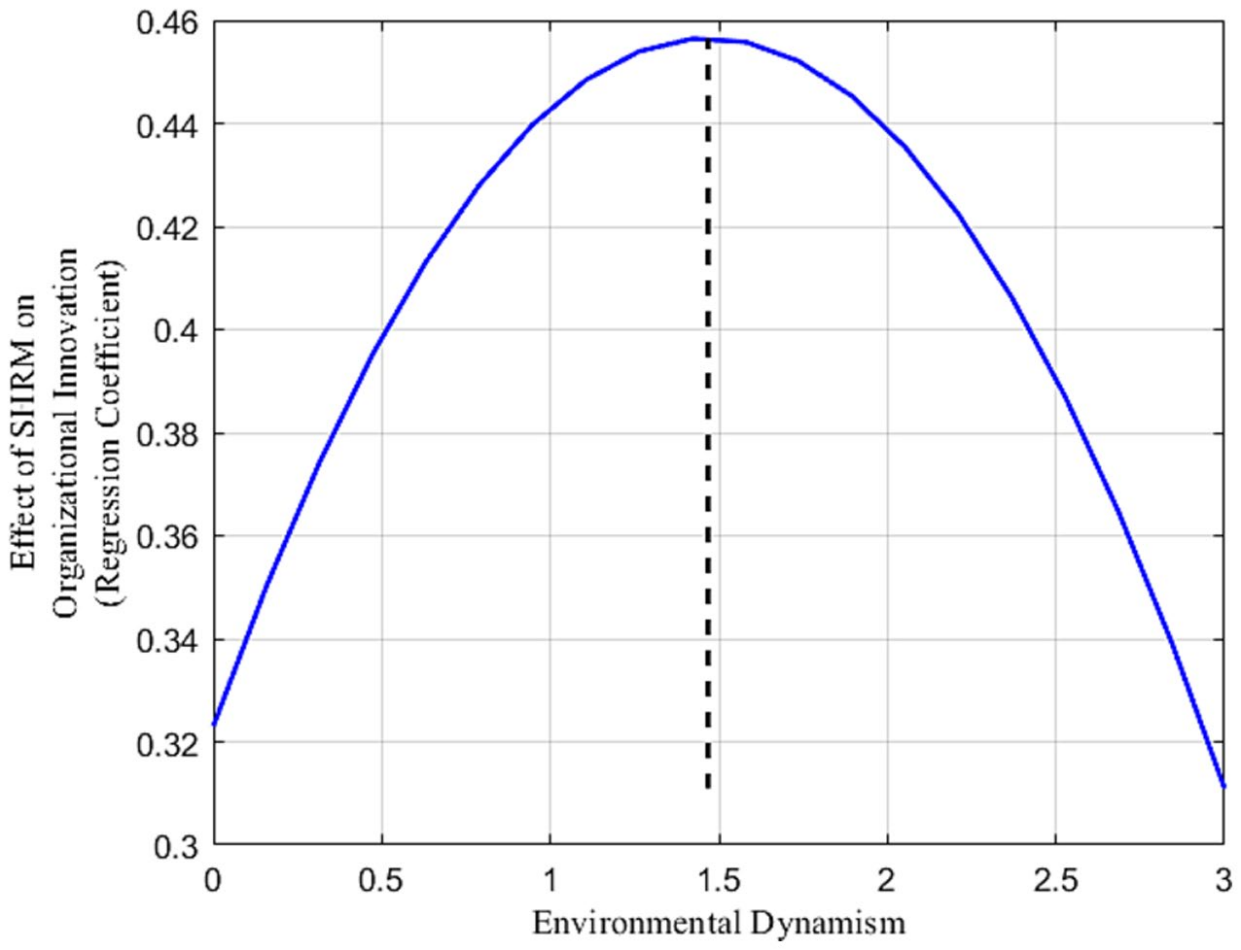
Inverted U-shaped moderating role of environmental dynamism in the relationship between SHRM and organizational innovation. Inflection point = 0.182/(2*0.062) = 1.468.

**Figure 4:**
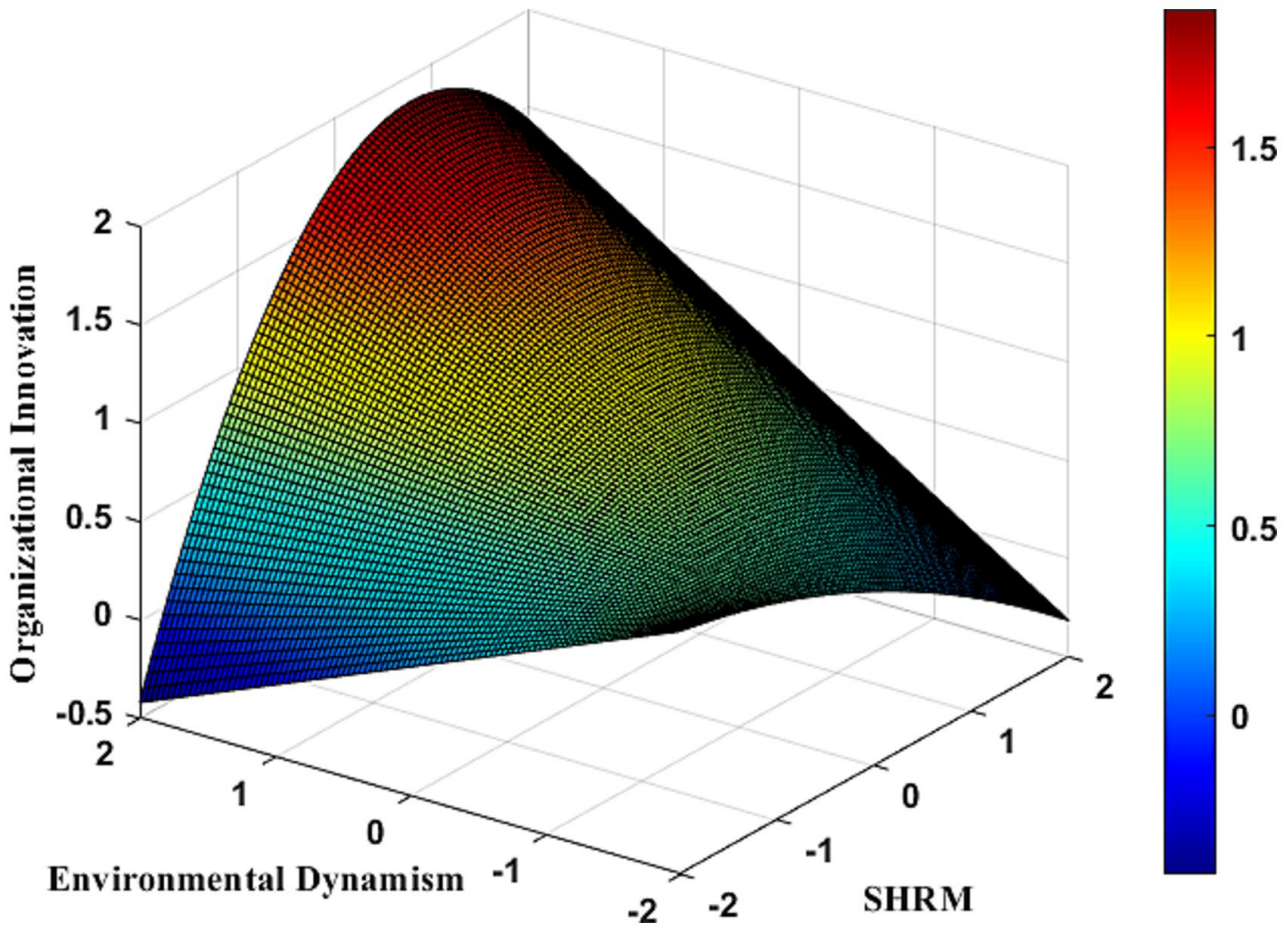
Three-dimensional inverted “U” shaped moderating role of environmental dynamism between SHRM and organizational innovation.

Table 7 presents the results of the moderating role of environmental dynamism on organizational innovation. In *Model 6c*, the interaction between SHRM and environmental dynamism shows a significantly positive regression coefficient (*b* = 0.243, *p* < 0.001). This indicates that SHRM significantly enhances organizational innovation in SMEs under moderate environmental dynamism. However, as environmental dynamism increases, the positive impact of SHRM on organizational innovation diminishes. Specifically, the quadratic interaction term between SHRM and environmental dynamism is significantly negative (*b* = −0.062, *p* < 0.001) in *Model 7c*, suggesting that beyond a certain threshold of environmental dynamism (inflection point = 1.468), excessive external changes can negatively impact the SHRM-organizational innovation relationship, disrupting the “learning-resilience” pathway in SMEs. To further explore this relationship under varying environmental dynamism levels, an inverted U-shaped moderating effect was visualized (see Figure 3). Table 9 demonstrates that at very low levels of environmental dynamism (M-2SD), SHRM negatively impacts organizational innovation in SMEs [*β* = −0.944, 95% CI (−1.213, −0.695)]. As environmental dynamism increases (M + 1SD), this impact turns positive [β = 0.454, 95% CI (0.297, 0.608)]. Notably, at higher levels (M + 2SD), the positive effect of SHRM on organizational innovation becomes insignificant [β = 0.251, 95% CI (−0.228, 0.709)]. This illustrates a nonlinear trend in SHRM’s impact on organizational innovation with increasing environmental dynamism. Figure 3 visually depicts this nonlinear pattern. To more intuitively demonstrate these nonlinear changes, Matlab 2021 software was used to generate (Figure 4). The three-dimensional interaction plot in Figure 4 further indicates that as environmental dynamism rises, SHRM’s positive impact strengthens but begins to weaken or turn negative beyond a certain threshold, reinforcing the inverted U-shaped moderating effect. This finding supports Hypothesis 6, confirming the influence of the “dynamic-changing” environment on organizational innovation in SMEs.

**Table 9:** Inverted U-shaped moderating role of environmental dynamism in the relationship between SHRM and organizational innovation.

	Environmental dynamism	Effect value	S. E.	95% confidence interval	
				Lower	Upper
SHRM→OI	M-2SD	−0.944	0.132	−1.213	−0.695
	M-1SD	−0.143	0.085	−0.323	0.012
	M	0.323	0.085	0.145	0.473
	M + 1SD	0.454	0.080	0.297	0.608
	M + 2SD	0.251	0.240	−0.228	0.709

SHRM: Strategic Human Resource Management; OI: Organizational Innovation.

## 5 Discussion and conclusions

Our study, grounded in the dynamic capabilities framework, explores the complex interaction between internal and external factors that shape the development of innovation capabilities in SMEs. We argue that SHRM plays a crucial role in enhancing organizational innovation, especially within SMEs. Implementing customized strategies related to employee recruitment, training, compensation, motivation, and participation—aligned with the innovative goals of SMEs—effectively harnesses the creative potential of human capital, thus promoting organizational innovation. This aligns with the recent work of Ho et al. (2023), who emphasized that SHRM practices tailored to innovation goals are critical for SMEs to leverage their human capital in dynamic environments. Organizational learning and resilience act as chain mediators in the positive relationship between SHRM and organizational innovation in SMEs. We suppose that these two elements are integral to strategic human resource allocation, serving as key internal drivers of innovation. This resonates with Youndt et al. (1996), who posit that human resource configurations directly influence organizational learning trajectories. Together, we establish a “*learning-resilience*” pathway that drives SMEs toward continuous innovation. Additionally, environmental dynamism influences the positive effects of SHRM on organizational innovation, following a nonlinear inverted “U”-shaped curve. Moderate environmental dynamism enhances learning capabilities within human capital, uncovers latent knowledge management skills, and strengthens SMEs’ resilience in the face of change, thereby reinforcing the foundation for organizational innovation. Conversely, excessive environmental dynamism, particularly its complexity, presents significant challenges to SMEs’ innovation efforts. These external uncertainties increase SMEs’ vulnerability, hinder the development of innovation capabilities among organizational members, and create a “dynamic-changing” environment that obstructs innovation activities. These results echo the contingency perspective of Simerly and Li (2000), who highlighted the threshold effects of environmental pressures on organizational outcomes. Our study reveals the evolutionary patterns of innovation capability development in SMEs under varying levels of environmental dynamism, providing fresh theoretical perspectives and practical insights for navigating and managing organizational innovation in the face of environmental uncertainty.

### 5.1 Theoretical implications

Our study makes significant contributions to theory in several ways. Firstly, in the ever-changing and tumultuous business environment, sustained innovation has become essential for enterprises, particularly SMEs, to navigate environmental shifts and ensure long-term survival. This research highlights the crucial role of SHRM in promoting organizational innovation within SMEs. By drawing on the dynamic capability view, we elucidate how and when SHRM can positively influence SMEs’ organizational learning and resilience, thereby enhancing their innovation performance. This aligns with Ferreira et al. (2020), who demonstrated that dynamic capabilities mediate the relationship between strategic practices and innovation. Our findings indicate that SHRM serves as a vital tool for SMEs to efficiently manage and reconfigure their human resources, thus fostering their innovative capabilities. This aligns with previous studies that emphasize the importance of HR practices in driving firm performance and innovation (Chen and Huang, 2009; Wright et al., 2001), but extends this understanding by focusing on the context of SMEs and the mediating roles of organizational learning and resilience.

Secondly, our study bridges the gap in existing literature by clarifying the chain mediating effects of organizational learning and resilience between SHRM and SMEs’ organizational innovation. By constructing an internal “*learning-resilience*” pathway, we contribute to the expanding field of organizational innovation research by demonstrating how SHRM fosters a learning culture that, in turn, builds organizational resilience, ultimately driving innovation. This finding supports the theoretical argument that learning and resilience are interlinked processes that collectively shape organizational adaptability and innovative capacity (Linden and Teece, 2014; Lengnick-Hall et al., 2011; Weick et al., 1999).

Finally, our study introduces a new understanding of the moderating role of environmental dynamism in the SHRM-innovation relationship within SMEs. We identify an inverted U-shaped moderating effect, suggesting that moderate levels of environmental dynamism stimulate SHRM activities, thereby enhancing organizational innovation and resilience. In contrast, excessive environmental dynamism impedes innovation efforts, reducing the positive impact of SHRM. This finding contributes to the broader discussion on the conditions under which external factors interact with internal capabilities to influence innovation performance (Simerly and Li, 2000; Jansen et al., 2009). Thus, our study advances the knowledge by illustrating how environmental dynamism shapes the effectiveness of SHRM practices in fostering innovation within SMEs (Jansen et al., 2009).

### 5.2 Practical implications

The practical implications of our study offer significant insights into enhancing the innovation capabilities of SMEs. Firstly, our findings emphasize the pivotal role of SHRM in positively influencing organizational innovation among SMEs (Chen and Huang, 2009). To translate SHRM investments into measurable performance outcomes, SME managers should conduct regular strategic human resource audits to align recruitment criteria, training content, and incentive systems with innovation KPIs (e.g., patent applications and time-to-market for new products). It is crucial for SMEs to prioritize the deployment of innovative human capital and increase investments in SHRM practices. As Sanders et al. (2021) noted, strategic HR configurations are pivotal in aligning human capital with innovation goals in turbulent environments. By doing so, SMEs not only establish a strong talent foundation for innovation but also better navigate turbulent and dynamic environments (Do et al., 2022), especially considering their limited resources and capabilities. For instance, adopting agile performance metrics that reward both exploratory learning (e.g., experimentation with emerging technologies) and exploitative learning (e.g., refining existing processes) can enhance innovation efficiency (Jansen et al., 2009).

Moreover, organizational learning and resilience emerge as key mediators in the relationship between SHRM and organizational innovation (Lengnick-Hall et al., 2011). This underscores the necessity for SMEs to cultivate a learning culture that promotes knowledge acquisition, sharing, and application across all organizational levels (Flores et al., 2012). Specifically, SMEs should establish institutionalized learning mechanisms, such as cross-departmental innovation task forces, post-project debriefing systems, and cloud-based knowledge repositories, to accelerate the conversion of individual expertise into organizational capabilities (Dyer and Nobeoka, 2000). As knowledge becomes the cornerstone of innovation, organizations that manage and leverage their knowledge resources effectively are better positioned to adapt and thrive amid environmental uncertainties (Nonaka, 2009). Additionally, fostering organizational resilience enables SMEs to swiftly recover from disruptions, maintaining operational efficiency and innovative momentum (Gittell et al., 2006). Practical interventions may include resilience training programs for employees (e.g., scenario planning workshops), redundancy design in critical skill sets, and dynamic reallocation protocols for human resources during crises (Lengnick-Hall et al., 2011).

Thirdly, our study also reveals that environmental dynamism moderates the relationship between SHRM and organizational innovation, suggesting that the positive effects of SHRM on innovation depend on the level of external environmental changes (Baik et al., 2019). To optimize this contingency effect, SME leaders should implement environmental scanning systems (e.g., AI-powered market trend analysis) and adopt modular HR architectures that allow rapid reconfiguration of teams and competencies when external turbulence exceeds predefined thresholds (Teece et al., 1997). As environmental dynamism intensifies, the influence of SHRM on innovation initially strengthens, indicating the need for SMEs to be agile and responsive. However, as environmental changes become overwhelming, the effectiveness of SHRM may diminish, highlighting the importance for SMEs to continually reassess and readapt their SHRM strategies to align with evolving market conditions. This finding emphasizes the need for SMEs to maintain a proactive stance, continuously scanning the external environment and flexibly adjusting their human resource configurations to remain competitive.

Lastly, our research highlights the dynamic balance between internal factors, such as organizational learning and resilience, and external environmental dynamism in shaping the innovation capabilities of SMEs (Eisenhardt and Martin, 2000). We recommend that SMEs establish innovation governance committees to systematically monitor the alignment between internal capability-building initiatives (e.g., monthly learning sprints) and external dynamism indicators (e.g., industry innovation cycles). This presents a practical framework for SMEs to effectively manage and leverage both internal and external factors to drive organizational innovation. It underscores the need for SME managers to adopt a contingency approach, tailoring their SHRM strategies according to specific environmental characteristics to maximize the development and enhancement of innovation capabilities. For example, in hypercompetitive sectors, combining decentralized decision-making authority with real-time knowledge-sharing platforms can enhance both learning speed and adaptive capacity (Pavlou and El Sawy, 2006). By doing so, SMEs can not only survive but also thrive in increasingly volatile and competitive markets.

### 5.3 Recommendation and solution

Based on our findings, we propose a three-pronged strategic framework to optimize the “learning-resilience” pathway in SMEs. First, adopt adaptive SHRM architectures by integrating Modular HR systems that combine stable core competencies (e.g., digital skills certification programs) with flexible peripheral elements (e.g., gig talent pools for emerging technologies). Utilize dynamic competency mapping powered by AI-driven workforce analytics to align skill development with innovation. Establish ambidextrous incentive structures that balance exploitation rewards (e.g., process improvement bonuses) and exploration rewards (e.g., equity stakes for Hackathon winners).

Second, institutionalize capability-building mechanisms by establishing Chief Learning Officer positions to oversee knowledge lifecycle management. Implement resilience stress tests via war-gaming market disruptions (e.g., rapid prototyping challenges under resource constraints) and develop cross-functional mobility programs to enhance systemic adaptability.

Third, environmental sensing infrastructure should be created using blockchain-enabled ecosystem monitoring dashboards to track partner/supplier innovation activities. Establish predictive analytics units specializing in scenario planning for technological discontinuities and leverage open innovation platforms to crowdsource solutions from distributed talent networks.

This framework enables SMEs to dynamically calibrate their human capital strategies across the innovation value chain while maintaining strategic flexibility.

### 5.4 Limitations and future directions

Despite the valuable insights derived from our study, several limitations should be acknowledged, providing pathways for future research. First, our study’s participators predominantly consist of high-tech SMEs in the Yangtze River Delta region of China, specifically within industries like biomedicine and software development. This industry-specific focus constrains the generalizability of the findings. Future studies are encouraged to expand the sample scope, encompassing SMEs from diverse sectors and regions to verify the robustness of the observed relationships (Zahoor et al., 2020). Second, the reliance on self-reported surveys from SME employees and managers poses potential measurement errors or biases, restricting causal inference. Adopting a multi-informant approach, which includes input from customers, suppliers, and industry experts, could significantly enhance data reliability and validity in subsequent research (Filatotchev and Nakajima, 2010; Zhang et al., 2024). Third, while this study investigates the moderating role of environmental dynamism on the relationship between SHRM and organizational innovation, it does not comprehensively explore how environmental dynamism influences the chain mediating effects of organizational learning and resilience, nor does it examine potential nonlinearities in these processes. Future research should delve into these intricate mechanisms to provide a holistic understanding of the interplay between internal organizational factors and external environmental dynamics (Filatotchev and Nakajima, 2010). Fourth, this study employs a dynamic capability framework to elucidate how SHRM leverages human resources to foster learning and resilience, thereby driving innovation. Future research might integrate alternative theoretical perspectives, such as leadership theories (Dinh et al., 2014) or organizational culture frameworks (Assoratgoon and Kantabutra, 2023), to capture the multifaceted impact of SHRM on innovation. Finally, our study identifies an inverted U-shaped moderating role of environmental dynamism on the SHRM-innovation relationship. Future research should further investigate such nonlinear moderations empirically, offering deeper insights into the boundary conditions shaping SME innovation.

## Data Availability

The original contributions presented in the study are included in the article/supplementary material, further inquiries can be directed to the corresponding author.
